# Impact of Antiphospholipid Syndrome and/or Systemic Lupus Erythematosus on the Long-term Adverse Cardiovascular Outcomes in Patients After Percutaneous Coronary Intervention: A Systematic Review and Meta-analysis

**DOI:** 10.1097/MD.0000000000003200

**Published:** 2016-03-25

**Authors:** Pravesh Kumar Bundhun, Kamini Devi Boodhoo, Man-Yun Long, Meng-Hua Chen

**Affiliations:** From the Institute of Cardiovascular Diseases (PKB, M-YL, M-HC), the First Affiliated Hospital of Guangxi Medical University, Nanning, Guangxi; and Department of Rheumatology (KDB), Xiangya Hospital, Central South University, Changsha, Hunan, China.

## Abstract

Antiphospholipid syndrome (APS) and systemic lupus erythematosus (SLE) are 2 rare autoimmune disorders which commonly affect women. Several previous studies showed APS to have been evolved from SLE. Secondary APS often coexists with SLE. One common feature relating these 2 diseases are the antiphospholipid antibodies, which are found in most of the patients with APS and in approximately 30% to 40% of patients with SLE, among which, about 10% develop APS. The leading cause of death in these patients is from cardiovascular disease due to accelerated atherosclerosis, which often progresses more rapidly, compared with the general population. However, the impact of APS and/or SLE on the cardiovascular outcomes in patients undergoing percutaneous coronary intervention (PCI) is controversial. Therefore, to solve this issue, we aim to compare the long-term (≥1 year) adverse cardiovascular outcomes after PCI, in patients with APS and/or SLE, and those without these disorders.

Medline and EMBASE databases were searched for studies comparing the long-term adverse cardiovascular outcomes between SLE and non-SLE, APS and non-APS, or SLE + APS and non-SLE + non-APS after PCI. We calculated odd ratios (OR) and 95% confidence intervals (CIs) for these categorical variables, and the pooled analyses were performed with RevMan 5.3.

Seven studies consisting of a total of 253,436 patients (568 patients in the experimental group and 252,868 patients in the control group) were included in this meta-analysis. During a follow-up period of ≥1 year, mortality and myocardial Infarction (MI) were significantly higher in the experimental group (OR 2.02, 95% CI 1.63–2.49, *P* < 0.00001 and OR 1.59, 95% CI 1.23–2.05, *P* = 0.0004, respectively). Major adverse cardiac events and repeated revascularization were also significantly higher in the SLE/APS group (OR 2.40, 95% CI 1.42–4.03, *P* = 0.001 and OR 2.59, 95% CI 1.26–5.31, *P* = 0.01, respectively).

Antiphospholipid syndrome and SLE are associated with significantly higher long-term (≥1 year) adverse cardiovascular outcomes after PCI. However, because of the limited number of patients and researches done, and due to a larger percentage of heterogeneity observed among several subgroups, this analysis may not generate a powerful result.

## INTRODUCTION

Antiphospholipid syndrome (APS) and systemic lupus erythematosus (SLE) are 2 rare autoimmune disorders which are somehow related to each other.^1^ Long ago, studies showed APS to have been evolved from SLE. When further research was done, APS was finally classified as primary and secondary APS.^2^ Secondary APS often coexists with SLE. One common feature relating these 2 diseases are the antiphospholipid antibodies (aPL antibodies), which are found in most of the patients with APS and in approximately 30% to 40% of patients with SLE, among which, about 10% develop APS.^3^

Atherosclerosis in such patients tends to occur more often and advances more rapidly compared with those patients in the general population, and finally results in the development of coronary artery disease (CAD) followed by acute coronary syndrome (ACS). Studies have shown that the leading cause of death from cardiovascular disease in these patients could be due to rapidly developing atherosclerosis, which could further be accelerated by these aPL antibodies.^4–5^

Percutaneous coronary intervention (PCI) is the most common invasive procedure performed in these patients with APS and SLE. However, the impact of APS and/or SLE on the outcomes in patients undergoing PCI is controversial. Hence, to solve this issue, we aim to compare the long term (≥1 year) adverse cardiovascular outcomes after PCI, in those patients with APS and/or SLE, and in those patients without these autoimmune disorders.

## METHODS

### Search Strategy

Beginning from November 2015, we searched Medline and EMBASE databases, for studies related to APS and/or SLE, and ACS by typing the words “APS and/or SLE and Acute Coronary Syndrome,” and also replacing the word “APS and SLE” by their full forms “Antiphospholipid Syndrome and Systemic Lupus Erythematosus.” To widen the search, the word “percutaneous coronary intervention” and its short form “PCI” were also used because only a few researches were published on the relation of APS or SLE with ACS. Because of its common relation with APS and SLE, the term “anticardiolipin antibodies (aCL)” has also been used to find relevant articles. Only articles published in English language were considered. Our search for articles came to an end in December 2015.

### Study Selection

#### Inclusion and Exclusion Criteria

Studies were included if:

1. they were randomized controlled trials or observational studies
2. they compared APS with non-APS or SLE with non-SLE or APS/SLE with non-APS/non-SLE in patients with ACS or patients who have undergone PCI. Comparing cardiovascular outcomes in patients with high aCL antibodies (IgG > 40) and low aCL (IgG < 40) antibodies were also considered in the inclusion criteria since a higher titer of aCL (IgG > 40) antibodies was observed in many of these patients with APS and SLE.

Studies were excluded if:

1. they were case studies or letters to editor
2. they were not related to patients with ACS
3. data for the control group were not provided
4. these studies did not consist of patients with APS, SLE, or with a high aCL titer
5. these studies included patients under the age of 18 years.

### Defining Important Terms, Endpoints, and Follow-up

Endpoints included the following:

1. Death: all-cause death
2. Myocardial infarction (MI): any type of MI or reinfarction or ischemia
3. Repeated revascularization including target vessel revascularization
4. Major adverse cardiac events (MACEs) indicated major adverse cardiovascular event (including death, MI, or need for repeated revascularization). Adverse vascular events including MI, stent or vascular occlusion, revascularization, deaths, and other MACEs were considered in this same section too.

Follow-up period included a long-term duration of 1 or more years (up to 2 years), or a much longer follow-up period of more than 3 years. The major reported endpoints and the duration of the follow-up periods of each of the included studies have been represented in Table 1.

**TABLE 1:**
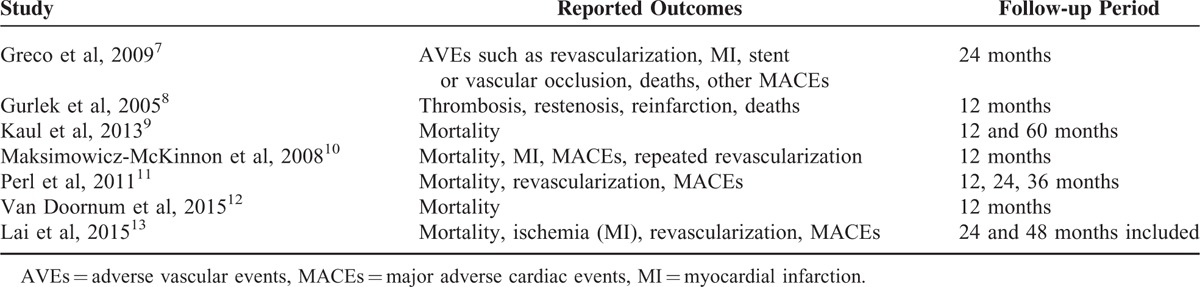
Show the Reported Outcomes and the Follow-up Periods of the Included Studies

Table 1 shows all the reported clinical endpoints reported in these studies. However, endpoints which were common in at least 2 studies, such as, mortality, MI, revascularization, and MACEs, were compared and analyzed. Since only 1 study reported thrombosis and restenosis, these endpoints were ignored in this meta-analysis. Also, because only 1 study had a follow-up period of 6 months, data for this short-term period were not considered.

### Data Extraction and Quality Assessment

Three authors (PKB, KDB, and MYL) independently reviewed the data, analyzed the types of studies, and assessed the eligibility and methodological quality of the articles included in this meta-analysis. Information regarding the author names, year of publication, the number of patients involved, type of diseases (SLE and/or APS) and baseline characteristics, intervention strategies, the cardiovascular outcomes, and follow-up periods was systematically extracted. Authors disagreed about including certain studies in the beginning. However, disagreements related to study inclusion were discussed between the authors, and if the authors still could not reach a decision, disagreements were finally resolved by the fourth author (MHC). The bias risk within the studies (whether having a low bias risk, moderate bias risk, or a high bias risk) was assessed with the components recommended by the Cochrane Collaboration.^6^

### Methodological Quality and Statistical Analysis

The Cochrane Q-statistic (*P* ≤ 0.05 was considered significant) and the I^2^-statistic were used to assess heterogeneity across the studies. An I^2^ value of 0% indicated no heterogeneity, an I^2^ value of less than 50% indicated mild or moderate heterogeneity, whereas larger values (above 50%) indicated increased heterogeneity. A fixed-effect model was used if I^2^ was <50%. However, if I^2^ was >50%, a random-effect model was considered. Funnel plots were assessed for publication bias. We calculated odd ratios (ORs) and 95% confidence intervals (CIs) for categorical variables, and the pooled analyses were finally performed with RevMan 5.3 software. All the authors had full access to and had taken full responsibility for the integrity of the data. All the authors have read and agreed to the manuscript as written and presented. Ethical committee or institutional review board approvals are not required for systematic reviews and meta-analyses.

## RESULTS

### Study Selection and Identification

**FIGURE 1:**
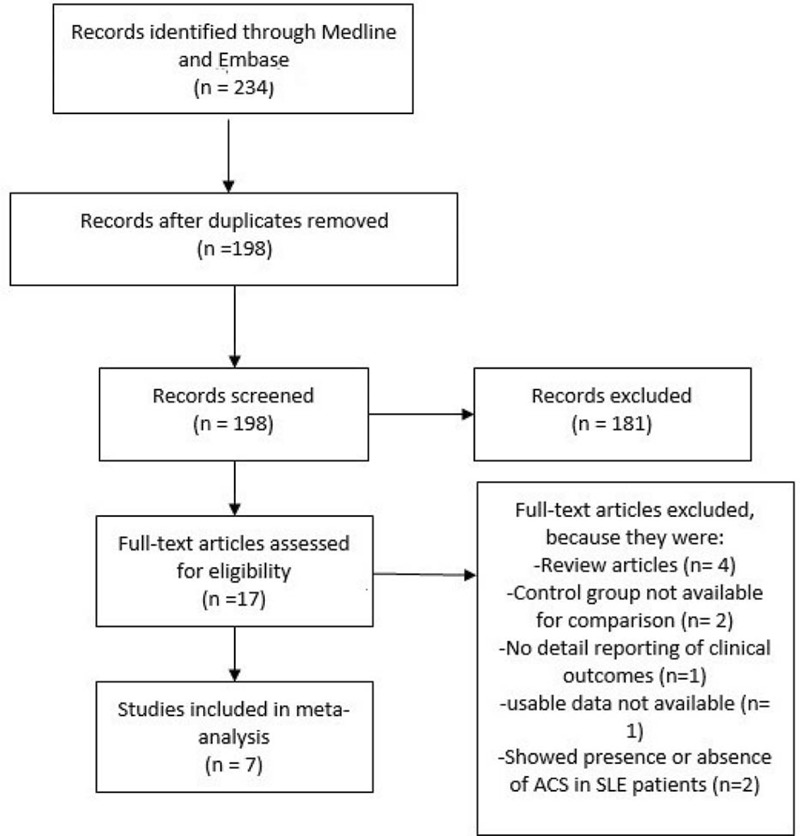
Flow diagram for the study selection.

In all, 234 articles were identified from Medline and EMBASE databases. After the elimination of duplicates and articles not related to our topic, 17 full-texted articles were finally assessed for eligibility. Among these 17 articles, 4 were review articles, 2 did not have a control group for comparison, 1 did not report the clinical outcomes in details, 2 only showed the presence or absence of ACS in these SLE or APS patients without comparing the adverse cardiovascular outcomes, and 1 study contained data not usable for our meta-analysis and therefore it was excluded. Finally, 7 articles which compared the long-term adverse cardiovascular outcomes after PCI among patients with APS and non-APS or SLE and non-SLE, or APS/SLE or non-APS/non-SLE were selected for this meta-analysis. The detailed flow diagram for the study selection has been represented in Figure 1.

### General Features of the Studies Included

A total of 7 studies consisting of 568 patients in the experimental group and 252,868 patients in the control group, with a total number of 253,436 patients, were included in this meta-analysis. The year of enrollment of patients, the regions where the corresponding researches were done, and the number of patients in the experimental and control groups, and also the diagnosis in these patients have all been listed in Table 2.

**TABLE 2:**
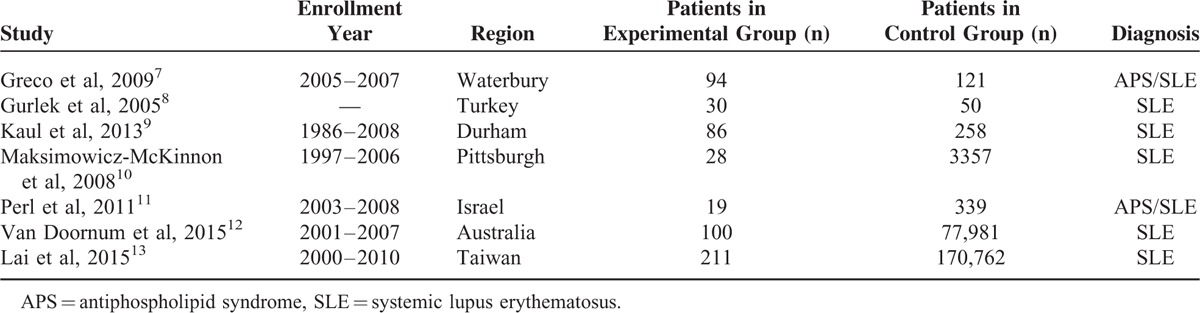
General Features of the Included Studies

One study enrolled patients between the year 1986 and 2008, whereas the other studies enrolled patients between the years 1997 and 2006, 2003 and 2008, 2005 and 2007, 2001 and 2007, and 2000 and 2010. One study did not report the enrollment period. There were more patients in the control group compared with the experimental group. General details of the 7 included studies have been shown in Table 2.

### Baseline Characteristics of the Included Studies

Table 3 shows the baseline features of the included studies.

**TABLE 3:**
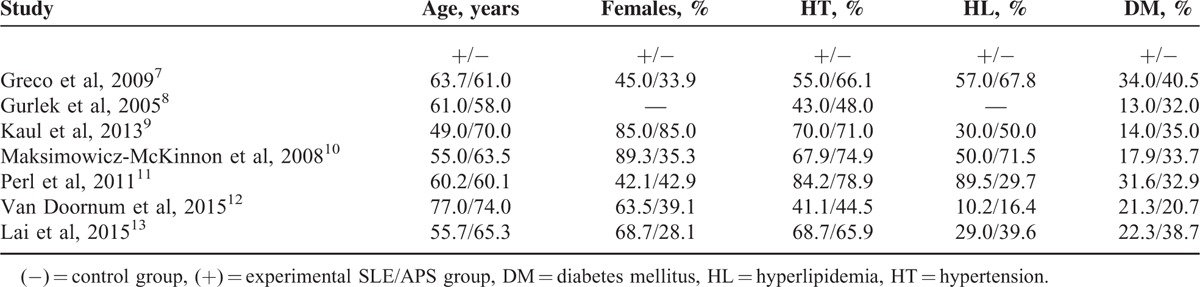
Baseline Features of the Included Studies

According to Table 3, 3 studies included younger patients in the experimental group. The percentage of female patients was similar in both the experimental and the control groups, except for 1 study, which consisted of 89.3% females in the experimental group and 35.3% in the control group. However, overall, there was no significant difference in the baseline characteristics between these 2 groups. If any difference existed, one study compensated for the other.

### Main Result of This Meta-analysis

**FIGURE 2:**
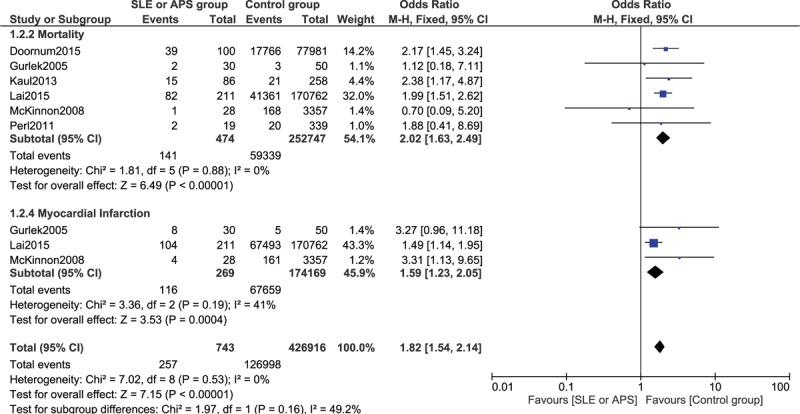
Forest plot showing the long-term results for mortality and myocardial infarction between the SLE/APS group and the control group after PCI. APS = antiphospholipid syndrome, PCI = percutaneous coronary intervention, SLE = systemic lupus erythematosus.

Results from this analysis showed that during a follow up of ≥1 year (less than 3 years), the adverse cardiovascular outcomes were significantly higher in the experimental group compared with the control group after PCI. A fixed-effect model was used to calculate the OR for mortality and MI during this follow-up period. Mortality and MI were significantly higher in the experimental group (OR 2.02, 95% CI 1.63–2.49, *P* < 0.00001 and OR 1.59, 95% CI 1.23–2.05, *P* = 0.0004, respectively). This result has been represented in Figure 2.

**FIGURE 3:**
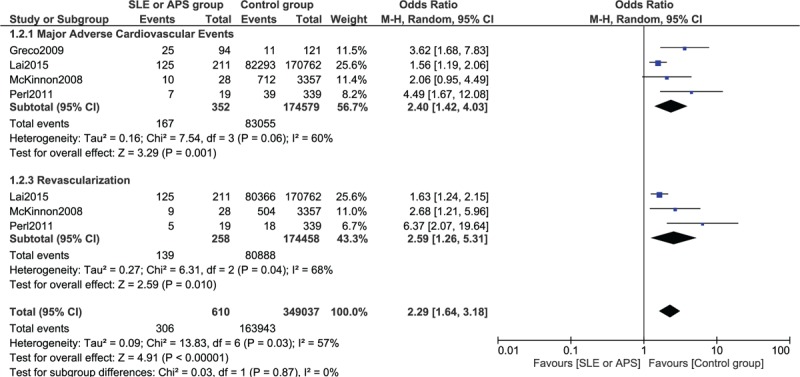
Forest plot showing the long-term results for major adverse cardiac events and repeated revascularization between the SLE/APS group and the control group after PCI. APS = antiphospholipid syndrome, PCI = percutaneous coronary intervention, SLE = systemic lupus erythematosus.

Since a higher heterogeneity was observed when calculating the OR for MACEs and repeated revascularization, a random-effect model was used. MACEs were significantly higher in the experimental group (OR 2.40, 95% CI 1.42–4.03, *P* = 0.001). Repeated revascularization was also significantly higher in the experimental group (OR 2.59, 95% CI 1.26–5.31, *P* = 0.01). This result has been shown in Figure 3. Table 4 shows the detailed overall results obtained when comparing the adverse cardiovascular outcomes between patients with SLE/APS and non-SLE/non-APS after PCI.

**TABLE 4:**
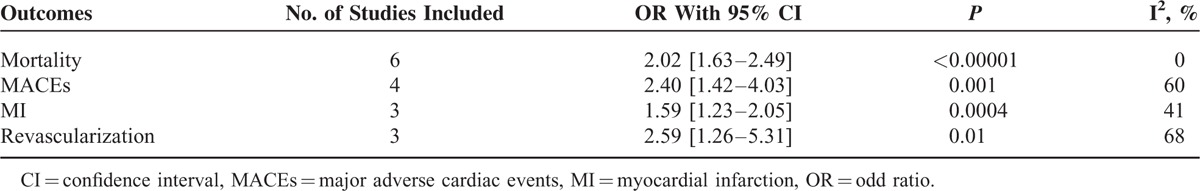
Results of This Meta-analysis

**FIGURE 4:**
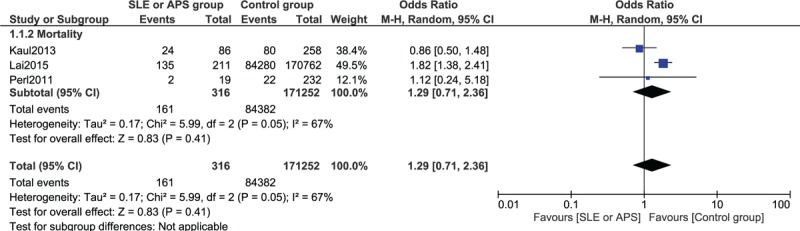
Forest plot showing the result for mortality after a follow-up period of 3 or more years after PCI. PCI = percutaneous coronary intervention.

For a longer follow-up period (3 years follow-up period or more), mortality rate was not significantly higher between the 2 groups (OR 1.29, 95% CI 0.71–2.36, *P* = 0.41). This result has been represented in Figure 4.

**FIGURE 5:**
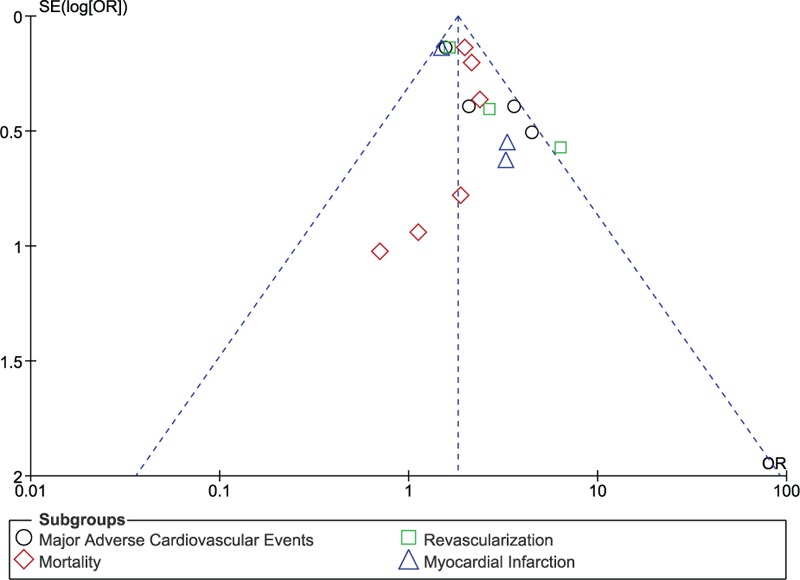
Funnel plot for sensitivity analysis.

For the above analyses, sensitivity analyses yielded consistent results. Based on a visual inspection of the funnel plot as mentioned previously in the “Methods” section, there has been no evidence of publication bias for the included studies that assessed the adverse cardiovascular endpoints. The funnel plot has been illustrated in Figure 5.

## DISCUSSION

Antiphospholipid syndrome and SLE, which are rare autoimmune diseases, affect a small population of women worldwide (less than 5%). These autoimmune disorders may probably be related to each other in one way or the other, for example, due to the presence of aPL antibodies.^14^ Patients suffering from these disorders are more at risk of suffering from an accelerated rate of progressive atherosclerosis which can finally result in ACS.^15^ However, the impact of APS and/or SLE on the adverse cardiovascular outcomes in patients after PCI is still controversial. Hence, a comparison of the long-term (≥1 year) cardiovascular outcomes between patients with APS and/or SLE and non-APS/non-SLE after undergoing PCI was performed to solve this issue.

Our results showed that, among the patients analyzed, adverse cardiovascular outcomes were significantly higher in the experimental group compared with the control group after PCI. Mortality, MACEs, MI, and repeated revascularization were all significantly higher in the APS/SLE patients compared with the control group after PCI.

Many reasons have been proposed for such a result in these patients with APS and SLE. Association of aPL antibodies and atherosclerosis has been established. A strong association between the levels of aCL antibodies and the outcomes of PCI after an ACS has also been observed.^8^

Several studies have reported elevated levels of aCL antibodies (which is normally present in patients with APS and SLE) in young patients who had survived a MI, and a higher rate of subsequent cardiovascular events occurred in these patients.^16^ Moreover, high-density lipoproteins (HDLs), a healthy cholesterol required by our body, were showed to be reduced in patients with active SLE. To suppress the immune system in such patients, corticosteroids are required. Treatment of these patients with corticosteroids result in an increase in low-density lipoproteins (LDLs) and triglycerides in the body which could then further accelerate the process of atherosclerosis.^17–18^ Moreover, the aPL antibodies found in these patients could potentially cross-react with antibodies against oxidized LDL due to similarities between cardiolipin, β_2_ glycoprotein I, and LDL molecules. In turn, antibodies against oxidized LDL could further accelerate the progression of atherosclerosis.^19^

Very few articles have been published on the effect of APS and SLE in patients after PCI. Previous studies have also shown a result almost similar to ours, showing rates of adverse clinical outcomes to be higher in APS and SLE patients, and these studies have linked levels of aCL antibodies with the increased risk of MI and cardiac deaths.^20^ Researches have shown a strong association between the levels of aCL antibodies and the adverse cardiovascular outcomes after PCI. The study by Perl et al in 2011 also showed a remarkably higher rate of repeated revascularization (target vessel revascularization) in patients with APS who underwent coronary angioplasty despite a similar use of drug eluting stents and diabetic status in both the experimental and the control group.^11^ The study by Smythe et al in 1976 showed an increased incidence of MI among patients with SLE, which resulted in an increased mortality rate among such patients. The study by Hak et al^21^ also concluded that they found a statistically increased risk of cardiovascular diseases among patients with SLE. Several other studies showed a higher prevalence of mortality in patients with conditions such as APS or SLE, and they also found a higher number of younger women who were associated with these conditions. Bruce et al^22^ confirmed the onset of CAD in a Toronto cohort at a younger age. In another review, Petri^23^ showed the prevalence of CAD in SLE patients to be between 6% and 54%, and mortality in these patients varied from 3% to 45%. Hence, higher adverse outcomes are most probably expected after PCI in these patients.

Our study did not compare the restenosis rate after PCI due to a lack of data for restenosis among the studies included. However, other studies have reported a significantly higher incidence of coronary restenosis in such patients. The study by Gurlek et al showed that a higher level of aCL antibodies predicted increased rate of restenosis after PCI.^8^ However, their study did not show any increase in mortality rate in the experimental group. Similarly, another study by Eber et al^24^ showed an increased rate of restenosis after percutaneous transluminal coronary angioplasty in such patients. Several different mechanisms were shown to be involved with high aCL-IgG levels and restenosis after PCI.^25^

However, whether this association of restenosis after PCI is strictly linked with SLE or APS is still considered a controversy. Patients without these autoimmune diseases, but with positive aCL antibodies, have shown to have a higher rate of restenosis after PCI showing a direct association of restenosis with these antibodies.^26^ Nevertheless, a study by Sharma et al^27^ failed to demonstrate any significant correlation between the level of IgG aCL antibodies and in-stent restenosis after PCI. Another study by Chiarugi et al^28^ observed no association between the presence of aCL and clinical restenosis; however, the presence of aCL with elevated lipoprotein a levels, acting synergistically, increased the risk of restenosis.

Our study reported adverse clinical outcomes in such patients (SLE and APS) after PCI. However, controversies also exist between the relation of aCL antibodies and adverse cardiovascular outcomes. Even if several studies have shown an increase in adverse outcomes, the study by Gurlek et al found no relation between aCL antibodies and recurrent cardiovascular events in ACS patients suffering from APS/SLE.^8^ Another study by Sletnes et al^29^ failed to prove that aCL is an independent risk for mortality or recurrent MI. Moreover, the study by Bruce^30^ showed SLE to be the main cause of death among all the SLE patients analyzed according to sex, age, race, and duration of illness, implying that SLE could be an independent cause of death in these patients.

This study has compared the adverse cardiovascular outcomes between patients with APS and/or SLE, and patients without APS and/or SLE after PCI. However, the presence of positive aCL antibodies in these SLE/APS patients is also associated with other catastrophic cardiac manifestations such as cardiomyopathy, valvular heart disease, and intracardiac thrombi, which are life threatening. These manifestations have been explained more clearly in other studies.

Only a few researches have been published, showing the association of adverse clinical outcomes in APS and SLE patients after PCI. Hence, our study is new since it is among the first meta-analysis comparing the long-term adverse clinical outcomes in such patients after PCI. Our study satisfies most of the requirements for a meta-analysis, in terms of low heterogeneity (except for MACEs and revascularization), absent publication bias, and sensitivity analysis, and provides robust scientific validity to our findings, which can assist informed decision-making by patients and physicians when predicting prognosis and treatments in such patients. However, due to the limited number of studies and patients, these data may not be sufficient to generate a good result. Moreover, because such patients often have restenosis and a higher rate of thrombosis, analysis of these clinical features is also important to generate a better conclusion.

### Limitations

This present study has several limitations. First of all, due to a limited number of researches concerning these patients and the smaller population size of APS and SLE patients used in our study, the results of this study could have been affected. Because of a limited number of studies in such patients after PCI, not much data were available to compare the different adverse outcomes between the 2 groups. Moreover, 1 among the 7 studies included, did not involve PCI, but only showed an association of SLE/APS with angiographically defined CAD. Another study dealt with the aCL antibodies, and a low level of less than 40 was considered as the control, whereas a high level of more than 40 was assumed to be in the experimental group during the comparison. However, not all patients with these antibodies will develop APS, or not all APS/SLE patients will have an increased aCL antibody level, because aCL antibodies have been found in about 5% of the healthy population too. Hence, this may have an effect on our result. Moreover, the study Kaul et al consisted of patients with an average age of 49 years in the experimental group and patients with an average age of 70 years in the control group. This could also be a main limitation which could have a large effect on our results. Also, because this study consisted mainly of observational studies, the bias risk may be higher, thus contributing as a limiting factor in this meta-analysis.

## CONCLUSIONS

Antiphospholipid syndrome and SLE have a greater impact on the long-term adverse cardiovascular outcomes in patients after PCI. Patients suffering from APS and/or SLE have a significantly higher rate of adverse cardiovascular outcomes after PCI. However, because of the limited number of researches and population size of patients in the experimental group, and due to the lack of data reporting restenosis and stent thrombosis, and also a higher percentage of heterogeneity observed among several subgroups, further researches including patients analyzed from randomized controlled trials will have to be conducted to completely solve this issue.
